# Reassessing sonography-based malignancy prediction in categories III and IV of previous Bethesda System for Reporting Thyroid Cytopathology in thyroidology

**DOI:** 10.1590/1806-9282.20250599

**Published:** 2025-12-05

**Authors:** Ilker Sengul, Demet Sengul

**Affiliations:** 1 Giresun University, Faculty of Medicine, Thyroidology Unit – Giresun, Turkey.; 2 Giresun University, Faculty of Medicine, Division of Endocrine Surgery – Giresun, Turkey.; 3 Giresun University, Faculty of Medicine, Department of General Surgery – Giresun, Turkey.; 4 Giresun University, Faculty of Medicine, Department of Pathology – Giresun, Turkey.

Dear Editor,

Thyroidology, Thyroid Cytopathology, and Neck-Endocrine Surgery remain comprising dynamic but challenging global issues, particularly in indeterminate cytology (IC). The novel 2023 Bethesda System for Reporting Thyroid Cytopathology (TBSRTC) has announced up-to-date recommendations with novel terminology in thyroidology and thyroid cytopathology^1–10^. I am writing to kindly suggest my comments on the recently published article entitled "Ultrasound Findings Suggestive of Malignancy in Thyroid Nodules Classified as Follicular Lesion of Undetermined Significance or Follicular Neoplasm based on the 2017 Bethesda System for Reporting Thyroid Cytopathology" by Jung et al.^11^. This study addresses a crucial challenge in thyroid nodule management: the preoperative risk stratification of nodules with Category III and IV of IC, The Bethesda System for Reporting Thyroid Cytopathology (TBSRTC) via investigating their sonographic features. For this purpose, a sum of 70 cases, 57 Category III, and 13 Category IV who had undergone surgery were incorporated, and sonographic findings were reviewed retrospectively.

Herein, the strengths of this study include the following points:

- The study focuses on a challenging diagnostic area by purposing to identify ultrasound (US) findings suggestive of malignancy in nodules classified as Category III and IV, based on the 2017 TRBSRTC, making accurate pre-operative risk stratification of this IC crucial,
- This research goes beyond standard assessments by evaluating additional US findings such as homogeneity of the solid portion, shape, and rim of the nodules, which are not typically the primary focus in systems like Korean Society of Thyroid Radiology Thyroid Imaging Reporting and Data System (K-TIRADS), in which exploration of novel features holds the potential to improve diagnostic accuracy,
- A key strength is the finding that these novel criteria demonstrated a significantly higher area under the curve (AUC) value compared to K-TIRADS, which suggests better overall diagnostic performance in predicting malignancy in this challenging group of nodules,
- The article provides a detailed statistical analysis of various US findings, including univariate and multivariate logistic regression, to identify malignancy predictors and evaluate the diagnostic performance [AUC, sensitivity, specificity, positive predictive value (PPV), negative predictive value (NPV), and accuracy] of individual US features and the developed criteria.

Nevertheless, the weaknesses of this valued study include the following points:

- The study's retrospective design introduces the potential for recall bias and limitations in data collection and interpretation,
- The relatively small sample size may limit the statistical power of the analyses and the robustness of the conclusions,
- Being a single-center study, the findings might not be directly generalizable to other populations or healthcare settings with different prevalences of thyroid diseases or variations in US equipment and expertise,
- The inclusion criteria, which focused on nodules that underwent surgical excision, likely led to a selection bias and a high overall malignancy rate, in which high prevalence might not reflect the typical distribution of benign and malignant nodules in the broader population of these cases, potentially might overestimating the predictive value of the identified US features in routine clinical practice,
- Noninvasive follicular thyroid neoplasm with papillary-like nuclear features (NIFTP) was classified as malignant in the statistical analysis. The current understanding considers NIFTP to be a low-risk entity, and its inclusion as malignant could have influenced the overall results and the assessment of diagnostic performance,
- Would the utilization of thicker or finer needles in order to obtain cytologic samples alter the outcome(s) of this study?^12–17^
- Would exerting the up-to-date 3rd edition of TBSRTC, considering both the novel subdivisions of category III, separately to all the studied and targeted purposes of this work, might affect the study's relevant outcome(s)?^4–8^

Consequently, while the study by Jung et al.^11^ provides valuable insights into potential US predictors of malignancy in thyroid nodules with this IC and recommends promising improvements over existing risk stratification systems, the aforementioned limitations necessitate cautious interpretation of the findings. As such, further prospective, multi-center studies with larger and more representative cohorts are needed to validate these findings and to assess their impact on clinical practice. Of note, special attention should be paid to the role of these sonographic features in different histological subtypes, including NIFTP, and to account for the variability in cytological diagnoses. To this end, further prospective studies with broader sample sizes from miscellaneous geographical regions, incorporating genetic and biological research, are essential to validate these findings and refine risk assessment strategies for thyroidologists. This issue merits further investigation. We thank Jung et al.^11^ for their worthy study on categories III and IV, the 2017 TBSRTC in thyroidology.

## Data Availability

The datasets generated and/or analyzed during the current study are available from the corresponding author upon reasonable request.
